# Process development and optimization of fed-batch production processes for therapeutic proteins by CHO cells

**DOI:** 10.1186/1753-6561-7-S6-P79

**Published:** 2013-12-04

**Authors:** Marie-Françoise Clincke, Mareike Harmsen, Laetitia Malphettes

**Affiliations:** 1 Cell Culture Process Sciences Group, BioTech Sciences, UCB Pharma S.A., Braine L'Alleud, Belgium

## Background

In the biopharmaceutical industry, process development and optimization is key to produce high quality recombinant proteins at high yields. As technologies mature, pressure on cost and timelines becomes greater for delivering scalable and robust processes. Overall, process development should be viewed as a continuum from the early stages up to process validation. Here we outline a lean approach on upstream development during the initial phases to optimize yields while maintaining the desired product quality profiles. Early-stage process development was designed to lead to the establishment of a baseline process and to systematically include experiments with input parameters that have a high impact on performance and quality. At this stage, potential for pre-harvest titer and yield increases as well as product quality challenges were identified. Feed adjustments and systematic experiments with top, high, and medium impact parameters have then been performed to develop a robust and scalable process. This approach was applied to two early stage upstream processes.

## Materials and methods

2L and 80L stirred tank bioreactors were run for 14 days in a fed-batch mode in a chemically defined medium. Feed was added daily from day 3 onwards. If required, antifoam C was added to the bioreactor by manual injections. DO, pH, and temperature were controlled at setpoint. DO was controlled using a multi-stage aeration cascade via a ring sparger. Viable cell concentration, cell viability, and average cell diameter were measured using a ViCell cell counter. The glucose, lactate, glutamine and ammonia concentrations were measured with a BioProfile Analyzer 400. On the day of harvest, the clarification was performed by centrifugation plus depth filtration. Monoclonal Antibody (MAb) concentration of the supernatant samples was quantified using Protein A high performance liquid chromatography.

## Results

**Figure 1:**
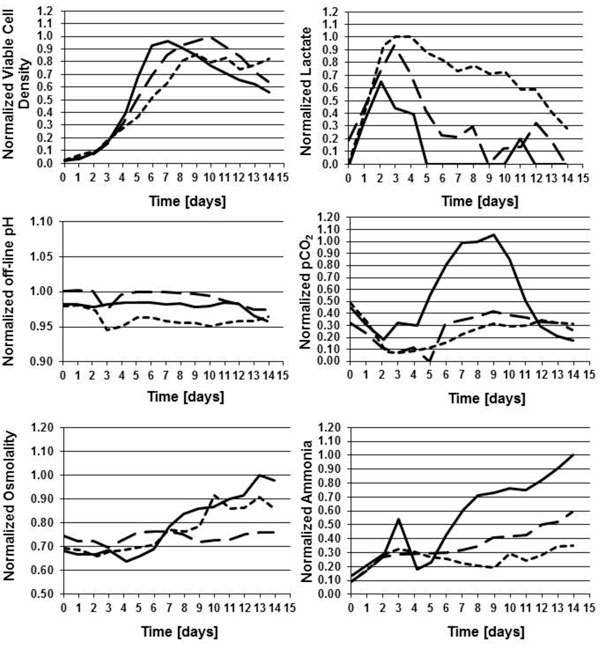
Viable cell concentration and off-line pH, pCO_2_, osmolality, lactate and ammonia profiles during fed-batch culture (solid black line: cell line 2, process 1 strategy, short dash line: cell line 1, process 1, long dash line: cell line 2, process 2)

A lean and Quality by Design (QbD) approach on process development during the initial phases to optimize yields while maintaining the desired product quality profiles was adopted. In this approach, a workpackage including the expected high impact parameters (feeding strategy, seeding density, pH, temperature and the interaction studies) was defined. This workpackage was applied to the process development of a cell line 1 producing a monoclonal antibody and led to a 36% increase in the monoclonal antibody titer compared to control condition (data not shown). Then, the operational process parameters and feeding strategy developed for cell line 1 (process 1) were applied to a cell line 2 producing a monoclonal antibody fragment. The application of the process 1 strategy to a cell line 2 was not the best for cell line 2 and led to high pCO_2 _level, high ammonia concentration, high osmolalities and low monoclonal antibody fragment titers (Figure 1). A feeding strategy was optimized for cell line 2 and pH set-point and deadband were also adjusted in order to decrease the pCO_2 _level. This optimized process for cell line 2 led to higher performances (pCO_2_, ammonia concentration, and osmolalities values were maintained at a low level) with a 43% increase in the monoclonal antibody fragment titer (data not shown). Then both processes were scaled up to 80L stirred tank bioreactors and comparable monoclonal antibody titers were obtained at 2L scale and 80L scale (Table 1). For the cell line 1, Product Quality Attributes such as Acidic Peak Group, aggregate and Mannose 5 were assessed and were maintained within the expected ranges with scale-up (data not shown).

**Table 1:** Comparison of MAb titers (normalized) obtained for both cell lines at 2L scale and 80L scale

	Cell line 1, Process 1	Cell line 2, Process 2
2L scale	1.00	1.00
80L scale	0.99	1.09

## Conclusions

A similar process development approach was applied to both projects where identical high impact parameters were identified. Although process optimized for cell line 1 was not the best for cell line 2, we were able to use it as a starting point and were able to optimize within the tight timelines. For both projects, high titers were achieved following our lean approach on process development. The final process 1 optimized for a cell line 1 led to a 36% increase in monoclonal antibody titer. The final process 2 optimized for a cell line 2 led to a 43% increase in monoclonal antibody fragment titer. Comparable titers and product quality attributes were observed at 2L scale and 80L scale. Hence the adopted feeding strategy proved to be robust and scalable.

